# Case report: Double-armed flanged polypropylene suture for repairing wide iridodialysis

**DOI:** 10.3389/fmed.2022.1084538

**Published:** 2023-01-12

**Authors:** Tingting Peng, Huafang Guo, Yong Wang, Li Zhou, Xianyi Bao

**Affiliations:** Aier Eye Hospital of Wuhan University (Wuhan Aier Eye Hospital), Wuhan, Hebei, China

**Keywords:** flange, double-armed, polypropylene, suture, iridodialysis

## Abstract

**Purpose:**

To describe a new technique for repairing wide iridodialysis (>180°) with a double-armed flanged polypropylene suture.

**Setting:**

Private practice, Wuhan, China.

**Design:**

Case report.

**Methods:**

Adjacent to the iridodialysis side, the sclera was punctured 2 mm exterior to the corneal limbus into the anterior chamber with a 30-G needle, then the root of the de-inserted iris was punctured. A 7-0 polypropylene thread was placed into the anterior chamber through a corneal incision on the opposite side and inserted into the needle. The needle was withdrawn, leaving one side of the suture out of the eye. Then, the sclera was punctured by a new needle 2 mm from the first puncture site and passed through the iris root 2 mm from the original iris puncture point. The other end of the thread was inserted into the needle and taken out of the eye. The suture was tightened to make the iris root adhere to the corneal limbus. Finally, the suture is was cut, and the ends were cauterized and left inside the sclera. This procedure can be repeated until the iridodialysis is solved.

**Results:**

The abovementioned technique was applied in four cases. At the end of the operations, the pupils of all patients were nearly round, with a diameter of about 3 mm. No patient suffered from intraoperative and postoperative complications.

**Conclusions:**

The double-armed flanged polypropylene suture is a simple and safe operation method that can be applied to repair wide iridodialysis.

## What was known

- The operation of iridodialysis repair is usually complicated. This article introduces a simple method.

## What this paper adds

- The double-armed flanged polypropylene suture is a simple method to repair wide iridodialysis.

## Introduction

Iridodialysis refers to a rupture at the junction of the iris and ciliary body caused by trauma or careless operation during intraocular surgery (1). It needed to be repaired when it appears in the lower half of the iris, is larger than one clock hour in the upper half, or induces double pupils because when left untreated, patients may suffer from visual problems, such as diplopia, photophobia, or cosmetic deformity (2). Different techniques for scleral fixation had been described (3); however, in iridodialysis >180°, surgery is usually troublesome, such as the sewing method undertaken using a sewing machine. The iris tissue was relatively soft and can be torn easily, so the suture technique needs to be minimally invasive and careful (4). In recent years, sutureless technique (5) or flange fixation (6) had been used to fix the intraocular lens in aphakic eyes. Notably, many iris suture techniques were similar to intraocular lens (IOL) suture techniques. Thus, we applied the double-armed flanged sutures to repair wide iridodialysis.

The studies involving human participants were performed according to the Declaration of Helsinki and approved by the Wuhan Aier Eye hospital's Ethics Committee (No. 2020IRBKY1201). Written informed consent was obtained from the individuals for the publication of any potentially identifiable images or data included in this article.

## Methods

**Figure 1:**
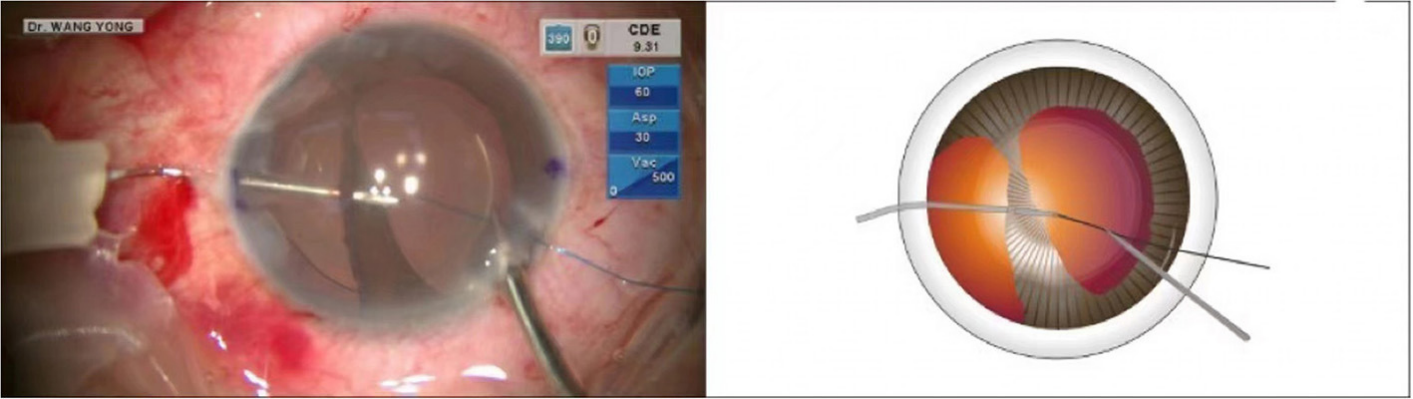
The needle was inserted into the anterior chamber from the sclera 2.0 mm behind the corneal limbus parallel to the iris, and the 23-G anterior forceps were used to assist the needle tip puncture through the root of the detached iris. Back off the needle and lead out one side of the suture that passes through the iris to the outside of the eye.

**Figure 2:**
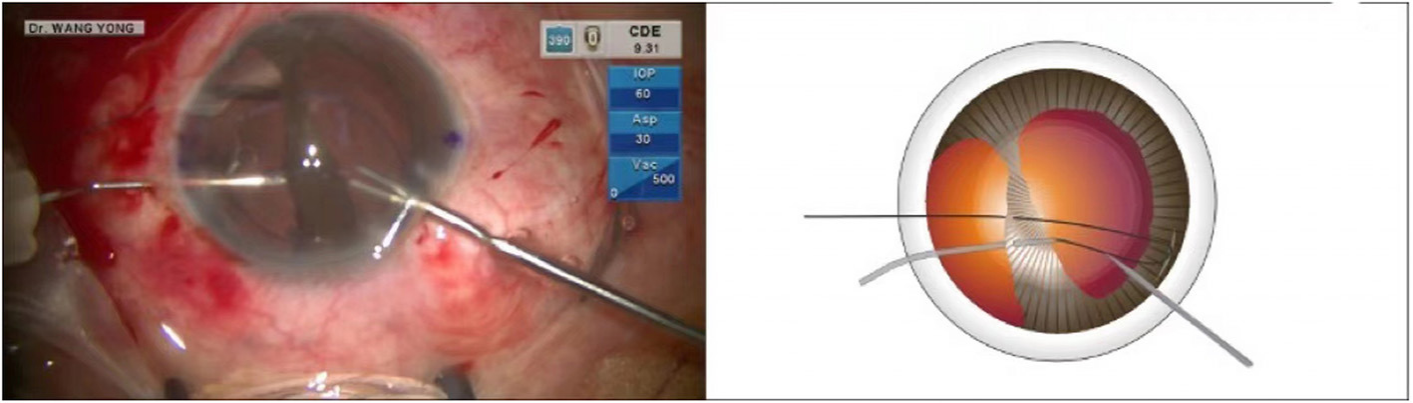
The needle was withdrawn, and the suture was taken out of the eye. The same procedure was repeated to guide the other arm of the suture through the iris and out of the eye.

**Figure 3:**
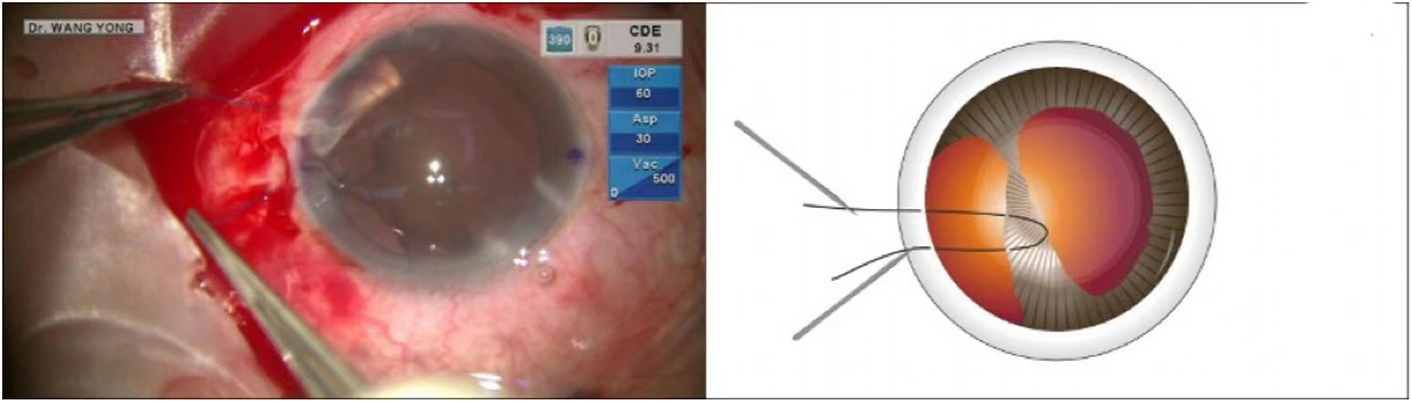
The suture was tightened to make the iris root adhere to the corneal limbus.

**Figure 4:**
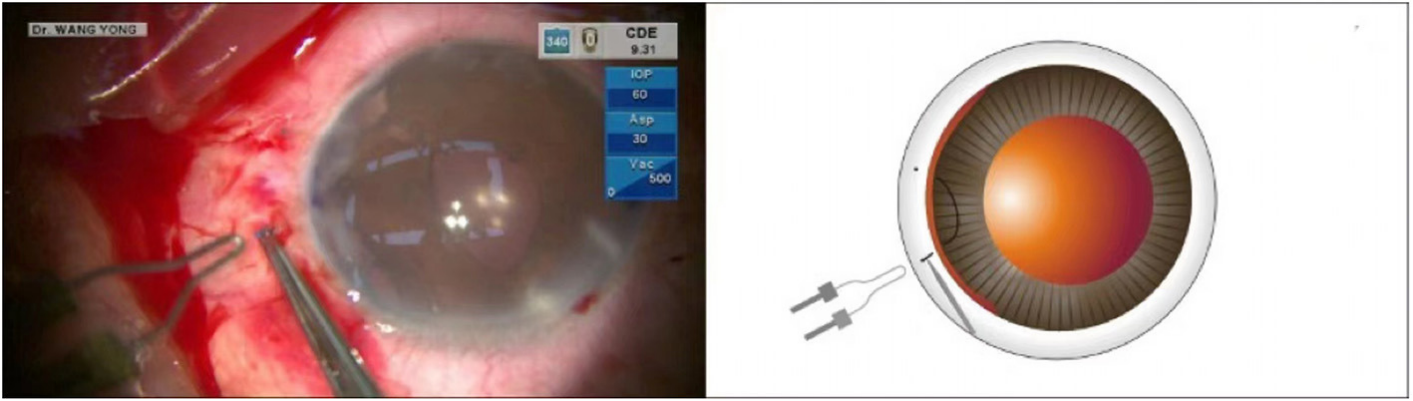
After cutting the suture, it was cauterized into a flange and inserted in the sclera for fixation.

This was a case of iridodialysis caused by injury during cataract surgery. The patient was a 52-year-old congenital cataract patient who underwent optical iridectomy 40 years ago. During cataract combined with pupillary plasty, due to operational complications, a large range of iridodialysis was caused. The operation of pupil repair was omitted in the surgical video, and only the operation of iris amputation repair was presented. The surgical video of iridodialysis repair can be viewed from the operation video (Supplementary Video 1). An angled sclerotomy was made through the conjunctiva using a 30-gauge (G) needle. The needle was inserted into the anterior chamber from the sclera 2.0 mm behind the corneal limbus parallel to the iris, and the 23 G anterior forceps (Belle Healthcare Technology Co, Ltd) were used to assist the needle tip puncture through the root of the detached iris, and the 7-0 polypropylene thread was clamped with 23 G forceps and inserted into the 30 G needle tube (Figure 1). Back off the needle and lead out one side of the suture that passes through the iris to the outside of the eye. The other side of the suture remains in the anterior chamber (Figure 2). The same procedure was repeated, and the other end of the suture was passed through the iris and out of the eye (Figure 3). The distance between both iris puncture points was 2 mm. In cases of wide dialysis, the same procedure was repeated until the repair was complete. Thereafter, the two ends of the same suture were tightened to facilitate adherence of the iris to the corneal limbus. The polypropylene sutures were then cut. Each end of the suture was flanged and shortened using high-temperature cautery until the flange reached the sclera (Figure 4).

## Results

The technique was applied in four cases (Table 1). Among them, 3 cases were iridodialysis combined with cataract and lens subluxation due to blunt ocular trauma, and 1 case was iridodialysis due to injury during cataract surgery. The range of dialysis was more than 180° in all patients.

**Table 1:** Thank you for your suggestion.

**Case**	**Sex**	**Age**	**Etiology of iridodialysis**	**Time interval from onset to treatment (months)**	**Range of dialysis**	**Management**	**Complications**
1	M	61	Blunt ocular trauma	6	180°	Iris suture combined cataract surgery	None
2	M	67	Blunt ocular trauma	12	180°	Iris suture combined cataract surgery	None
3	M	55	Blunt ocular trauma	8	180°	Iris suture combined cataract surgery	None
4	F	52	Complication in cataract surgery		180°	Iris suture	None

Related references have been added to this article.

At the end of the operation, the pupils of all patients were nearly round, with a diameter of about 3 mm. No patient suffered from intraoperative and postoperative complications, such as hyphema, suture exposure, posterior synechia of the iris, or high intraocular pressure. No visual impairments of monocular diplopia and glare were observed postoperatively.

## Discussion

The repair of wide iridodialysis had always been a challenging procedure. At present, sewing machine suture technology is the main method utilized for repairing wide iridodialysis (7). This technique entailed conducting conjunctival dissections and making long scleral tunnels, which may cause severe damage and increase astigmatism. In addition, the sewing technology requires the needle to enter the anterior chamber repeatedly. The operation was complex, and the learning curve is long.

In recent years, some studies had reported on the technique of minimally invasive suture of iridodialysis. Kusaka et al. (8) described a technique for iridodialysis repair using intrascleral fixation of a 6-0 polypropylene suture with a flanged tip. The end of the suture was flanged with thermoplasticity to pinch the iris. The exterior suture was passed intrasclerally for fixation with the aid of the attached needle. This technique does not require conjunctival dissections. The flange end of the suture is used to fix the iris tissue. However, the iris tissue was very soft, and when the iridodialysis is large and the tension is high, it may be loosened.

In our cases, we described a modified double-armed flanged suture technique. This technique is similar to the flange IOLs fixation reported by Yamane et al. (9). A 30-G needle was used to guide a 7-0 polypropylene thread to puncture the iris, and both arms of the suture were led out of the eye through the needle. Instead of the previous suture, one side flange was outside the eye, while one was in the iris. As iridodialysis was wide and the iris is soft, the intraocular flanged tip used to pinch the iris may be loosened. As the double flanges of the suture were on the extraocular scleral surface, the loosening of the suture was avoided. This method does not require the conjunctiva to be cut and sutured. For a wide range of iridodialysis, the suture operation can be repeated two to three times at an interval.

In summary, the use of a double-arm flange with an intrascleral polypropylene suture for repairing wide iridodialysis was an easy and safe treatment.

## Data availability statement

The original contributions presented in the study are included in the article/Supplementary material, further inquiries can be directed to the corresponding authors.

## Ethics statement

The studies involving human participants were performed according to the Declaration of Helsinki and approved by the Wuhan Aier Eye hospital's Ethics Committee (No. 2020IRBKY1201). Written informed consent was obtained from the individuals for the publication of any potentially identifiable images or data included in this article.

## Author contributions

All authors listed have made a substantial, direct, and intellectual contribution to the work and approved it for publication.
